# Airpod Sign: A Diagnostic Radiological Finding of a Rare Cerebrovascular Accident

**DOI:** 10.7759/cureus.32129

**Published:** 2022-12-02

**Authors:** Aiswarya Raj, Paul J Alapatt, Arya R, Sreevidya L K, Ashraf V V

**Affiliations:** 1 Department of Neurology, Aster Malabar Institute of Medical Sciences (MIMS), Kozhikode, IND

**Keywords:** neuro radiology, stroke, posterior circulation stroke, medial medullary syndrome, bilateral medial medullary infarction

## Abstract

Quadriplegia or dysesthesia in all four limbs may be the initial symptom of bilateral medial medullary infarction (MMI), a very rare cerebrovascular accident with a dismal prognosis. Clinical diagnosis of bilateral MMI is still challenging and can be confirmed by diffusion-weighted (DW) magnetic resonance imaging (MRI) in the early stage. Here, we report the case of a 60-year-old male who presented to the emergency department complaining of numbness in all four limbs. DW-MRI was used to identify brain lesions 24 hours after the symptom onset. The infarct, on axial MRI sections, showed the characteristic ‘airpod sign’/heart-shaped appearance due to the morphology of the area involved in the medulla.

## Introduction

Medial medullary infarcts are typically unilateral and only very rarely bilateral. They are an incredibly uncommon kind of stroke presentation, making up less than 0.5% of all cerebral infarcts [1,2]. Medial medullary infarcts usually develop acutely or with a subacute onset, presenting with dysarthria, dysphagia, and quadriplegia, sometimes complicated by respiratory disorder, often mistaken in the initial stages for Guillain-Barré syndrome, and therefore has a delayed confirmation of diagnosis by imaging. Consequently, the prognosis of such patients is usually poor [3,4]. The heart-shaped infarct seen on magnetic resonance imaging (MRI) created by the unusual vascular supply of the medial medulla oblongata can be used to diagnose bilateral medial medullary infarction (MMI) [1]. The appearance of this heart-shaped infarct on diffusion weighted imaging (DWI) is also referred to as the ‘airpod sign’, for its peculiar morphology and resemblance to the well-renowned Apple AirPods. In the case described here, a patient had a bilateral medial medullary infarct, and DW-MRI of the brain was used to detect the infarcted area with its distinctive heart-shaped morphology. This case is therefore reported to illustrate the importance of early DW-MRI in potentially reducing mortality and morbidity of a rare cerebrovascular accident.

## Case presentation

**Figure 1:**
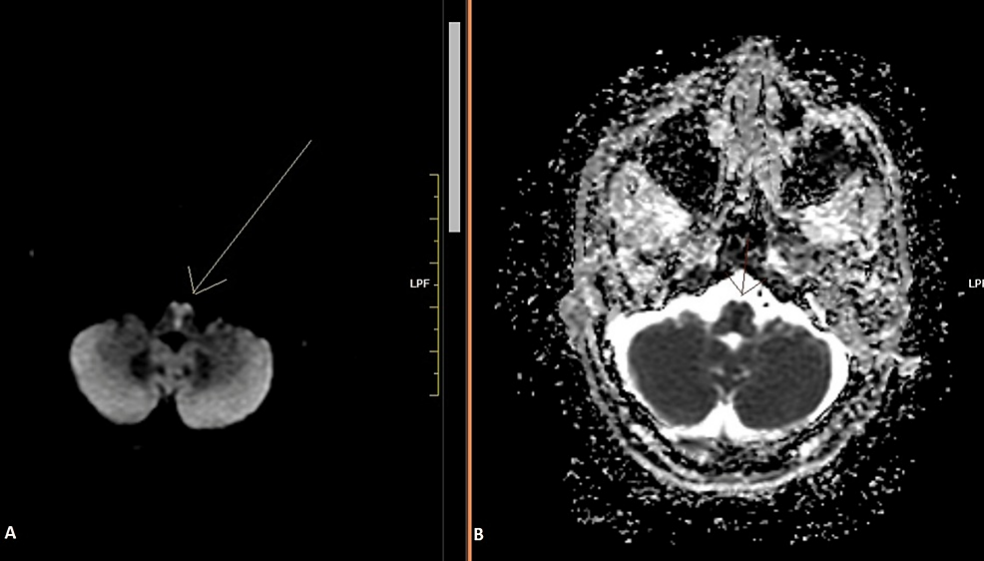
(A) A T2-weighted image showing the characteristic heart-shaped hyperintensity/airpod sign in the medulla, contrasted with (B) a corresponding drop in the apparent diffusion coefficient

A 60-year-old male, who had been previously diagnosed with type 2 diabetes mellitus and was on metformin, presented to the Emergency Department of Aster Malabar Institute of Medical Sciences (MIMS), Kozhikode, on August 10, 2022, at 4:30 pm, complaining of numbness in all four limbs since 10 am that morning. He also complained of giddiness and had had one episode of vomiting. His heart rate was 72 beats per minute, blood pressure 180/90 mmHg, and his body temperature was 36.6 °C when admitted. He had a Glasgow Coma Scale score of 15/15. There were no signs of any cranial nerve involvement. Pupils and eye movement were normal. There was no evidence of facial asymmetry or hearing impairment or asymmetry of the soft palate. Tongue protrusion was possible and there was no deviation on protrusion or weakness. The power assessed was found to be 5/5 on the Medical Research Council (MRC) Scale for Muscle Strength in all four limbs. All deep tendon reflexes were normal. However, he had bilateral lower limb incoordination, and due to this, he found it difficult to walk. Paraesthesia was noted over all four limbs. An initial screening brain MRI scan was done that did not show any acute infarction and was normal except for chronic age-related atrophy. Screening MRI of the cervical spine was also done at the time, to rule out any spinal pathology, which was normal. Routine blood investigations, electrocardiogram, and echocardiogram were normal as well. He was started on antiplatelets; however, by 9 am the next day, his power worsened to grade 0, and therefore, a repeat MRI brain scan was carried out immediately, almost 24 hours after the onset of his symptoms. DWI showed heart-shaped hyperintensity in the bilateral ventral medulla, with a corresponding hypointensity in the apparent diffusion coefficient (ADC) image, suggestive of the acute bilateral medial medullary infarct (Figure 1).

An MR angiogram was done to look for an underlying pathology of vertebral and basilar arteries and it came out normal. He was treated with antiplatelets, anticoagulants, IV antibiotics, and statins. He developed respiratory distress and had to be intubated and mechanically ventilated. Given the need for prolonged ventilatory support, a tracheostomy was done. The patient was gradually weaned off the ventilator to bilevel positive airway pressure (BiPAP) via tracheostomy and then to minimal O_2_ support. He had aspiration pneumonia during the hospital stay that was treated with IV antibiotics and nebulizations. He was started on chest and limb physiotherapy. Subsequently, the quadriplegia improved slightly with a power of 1/5 in both his lower limbs. However, power continued to be 0/5 in both his upper limbs. He was then transferred to a local hospital for inpatient rehabilitation and continuation of treatment under a physiatrist.

## Discussion

Clinical manifestations of bilateral MMI include hypoglossal nerve palsy, flaccid tetraplegia sparing the face, bilateral loss of deep sensation, and respiratory failure [4]. The typical signs associated with the medial medullary syndrome of Dejerine include (1) hemiparesis sparing the face contralateral to the infarct, (2) tongue weakness ipsilateral to the infarct, and (3) hemisensory loss of the posterior column type. These signs are attributed to the involvement of the area rostral to the decussation of the pyramidal tracts, the tracts and nucleus of the 12th cranial nerve, and the medial lemniscus [5,6]. Studies have shown that out of all these features, limb weakness is the predominant symptom in most, and gaze-evoked nystagmus was also a frequent finding [2].

Advances in neuroimaging have helped to arrive at a confirmed diagnosis of medial medullary infarct during the life of a patient [2]. Bilateral MMI, however, often presents as an acute onset tetraplegia and may often be misdiagnosed as Guillain-Barré syndrome [7]. Flaccid tetraplegia sparing the face was seen in the majority of reported instances. Infarct-related tongue weakness and sensory abnormalities are rather uncommon [8]. A systematic review by Pongmoragot et al. showed that the most frequent clinical manifestations were motor weakness (78.4%), dysarthria (48.6%), and hypoglossal palsy (40.5%), all of which were consistent with rostral medullary lesions. Atherosclerosis in the vertebral arteries accounted for 38.5% of all vascular pathologies, and poor clinical results were observed (mortality, 23.8%; dependence, 61.9%) [9].

Clinical diagnosis of bilateral medial medullary infarction is extremely difficult, and among the patients of this already rare cerebrovascular accident, there have been only around 15 cases of bilateral MMI that were confirmed by MRI [7]. In two of these cases, brain MRI was carried out 10 to 35 hours after the onset of symptoms, and diffusion-weighted imaging was used to confirm the infarction. In contrast, in the majority of the other cases, brain MRI was not carried out until several days after the onset of symptoms or hospitalization, and cerebral infarcts were only confirmed by T2-weighted imaging. In a similar case reported by Tokuoka et al., nine hours after the commencement, brain MRI was conducted, and DW-MRI was able to identify the infarcted area [7]. In our patient, the initial screening MRI did not show any abnormalities; however, the repeat MRI sequence performed 24 hours after the onset of symptoms showed the characteristic ‘airpod sign/heart-shaped infarct’ on DWI consistent with our provisional diagnosis of bilateral medial medullary infarct.

With regard to the underlying risk factors for the development of this rare stroke syndrome, according to Toyoda et al., a substantial association between atherosclerosis and MMI is seen, with hypertension, diabetes mellitus, and hypercholesterolemia being more frequently related than embolism [2]. Our patient also was a known case of type 2 diabetes mellitus, which likely contributed to his condition. However, their study showed that atherosclerosis of the vertebral artery, especially at its terminal portion, was the predominant underlying vascular lesion associated with MMI. MR angiography done to rule out such an etiology in our patient did not show any vascular abnormality, and the echocardiogram did not show any possible origin for a cardioembolic stroke. Conventional angiography often fails to demonstrate the visualization of the anterior spinal artery, and we postulate that a chronic atherosclerotic occlusion of the anterior spinal artery due to long-standing diabetes was the cause of this patient's infarction [2].

Penetrating arterioles from the anterior spinal artery to the caudal medulla and anteromedial arteries from the distal vertebral artery or proximal basilar artery to the rostral medulla supply the medial medullary territory, respectively [10]. According to an initial hypothesis, an abnormal branch of the vertebral artery may serve both sides of the medial medullary region, and its occlusion could cause simultaneous bilateral medullary infarcts [8]. Considering the high prevalence of anomalous vessels in this area, and the long-standing history of diabetes mellitus in our patient, which could lead to chronic atherosclerotic changes in small vessels, the occlusion of such vessels was most likely the underlying mechanism in our patient.

As was seen in our patient, who had to be intubated and put on ventilator support, respiratory failure frequently complicates these patients' inpatient stays, with a clear delay between the development of neurological symptoms and the onset of respiratory issues [2].

## Conclusions

The clinical diagnosis of bilateral medial medullary infarction is often extremely difficult, and this stroke syndrome is usually associated with a poor prognosis. Early confirmation of the clinical diagnosis using diffusion-weighted MRI is important, with the infarct showing a characteristic heart-shaped appearance/‘airpod sign’ on horizontal sections. Early diagnosis and treatment may help improve the long-term clinical outcome in such patients.
